# Editorial on the Topic “New Research on Detection and Removal of Emerging Pollutants”

**DOI:** 10.3390/ma16020725

**Published:** 2023-01-11

**Authors:** Avelino Núñez-Delgado, Zhien Zhang, Elza Bontempi, Mario Coccia, Marco Race, Yaoyu Zhou

**Affiliations:** 1 Department of Soil Science and Agricultural Chemistry, Engineering Polytechnic School, University Santiago de Compostela, 27002 Lugo, Spain; 2 Department of Chemical and Biomedical Engineering, West Virginia University, Morgantown, WV 26506, USA; 3 INSTM (National Interuniversity Consortium of Materials Science and Technology), Department of Mechanical and Industrial Engineering, University of Brescia, Via Branze, 38, 25123 Brescia, Italy; 4 Research Institute on Sustainable Economic Growth, National Research Council of Italy (CNR), Turin Research Area of the CNR, 10135 Turin, Italy; 5 Department of Civil and Mechanical Engineering, University of Cassino and Southern Lazio, Via Di Biasio 43, 03043 Cassino, Italy; 6 College of Resources and Environment, Hunan Agricultural University, Changsha 410128, China

With the Topic “New Research on Detection and Removal of Emerging Pollutants” (https://www.mdpi.com/topics/Emerging_Pollutants) closed to new submissions, the Editors would like to share some comments on it.

The journals involved in the Topic were Materials (with 23 papers finally published), Processes (with 21 papers published), Sustainability (with 13 papers published), Applied Sciences (with 7 papers published), and Toxics (with 2 papers finally published).

To date, with the Topic just closed for submissions, the most cited papers have received 22 citations [1], 21 citations [2], 15 citations [3], 10 citations [4], 9 citations [5,6], 8 citations [7], 7 citations [8,9], and 6 citations [10,11,12], while the other papers included in the Special Issue received between 5 and 0 citations at the time of writing of this editorial piece.

Overall, the Editors think that the Topical Issue has provided very interesting and high-quality contributions to the broad field of research on emerging pollutants. The removal of emerging pollutants is a challenging topic that is receiving increasing attention at the level of investigation and risk concern perceived by the society. In fact, improving the means for both quantification and removal of toxic substances is clearly relevant in the current situation of environmental stress affecting the different environmental compartments [13,14,15,16,17].

In addition, the Editors consider useful the experience of combining the five journals involved in the Topic which promotes a wider diffusion of this Special Issue, covering a broader spectrum of researchers and potential readers.

This field of research needs continuous and higher efforts, so it is expected that additional issues and Topics focused on it will be developed in the coming future.
